# Altered sensory feedbacks in pianist's dystonia: the altered auditory feedback paradigm and the glove effect

**DOI:** 10.3389/fnhum.2013.00868

**Published:** 2013-12-17

**Authors:** Felicia P.-H. Cheng, Michael Großbach, Eckart O. Altenmüller

**Affiliations:** Institute of Music Physiology and Musicians' Medicine, Hanover University of Music, Drama, and MediaHannover, Germany

**Keywords:** musician's dystonia, altered auditory feedback, glove effect, sensory trick, scale paradigm, sensorimotor integration

## Abstract

**Background:** This study investigates the effect of altered auditory feedback (AAF) in musician's dystonia (MD) and discusses whether AAF can be considered as a sensory trick in MD. Furthermore, the effect of AAF is compared with altered tactile feedback, which can serve as a sensory trick in several other forms of focal dystonia.

**Methods:** The method is based on scale analysis (Jabusch et al., 2004). Experiment 1 employs synchronization paradigm: 12 MD patients and 25 healthy pianists had to repeatedly play C-major scales in synchrony with a metronome on a MIDI-piano with three auditory feedback conditions: (1) normal feedback; (2) no feedback; (3) constant delayed feedback. Experiment 2 employs synchronization-continuation paradigm: 12 MD patients and 12 healthy pianists had to repeatedly play C-major scales in two phases: first in synchrony with a metronome, secondly continue the established tempo without the metronome. There are four experimental conditions, among them three are the same AAF as in Experiment 1 and 1 is related to altered tactile sensory input. The coefficient of variation of inter-onset intervals of the key depressions was calculated to evaluate fine motor control.

**Results:** In both experiments, the healthy controls and the patients behaved very similarly. There is no difference in the regularity of playing between the two groups under any condition, and neither did AAF nor did altered tactile feedback have a beneficial effect on patients' fine motor control.

**Conclusions:** The results of the two experiments suggest that in the context of our experimental designs, AAF and altered tactile feedback play a minor role in motor coordination in patients with musicians' dystonia. We propose that altered auditory and tactile feedback do not serve as effective sensory tricks and may not temporarily reduce the symptoms of patients suffering from MD in this experimental context.

## Introduction

Dystonia in pianists belongs to a group of dystonic movement disorders termed focal hand dystonias (Chen and Hallett, 1998). It is characterized by the degradation of voluntary control of highly skilled movement patterns involved in piano playing. The condition frequently results in co-contraction of wrist flexors and extensors and in involuntary curling, or extending of digits, thus rendering fast movements involved for example in scale playing irregular (Jabusch et al., 2004). It is known that focal hand dystonia involves several sensory abnormalities, such as reduced two-point discrimination thresholds, reduced graphaesthesia (Byl et al., 1996), and an impairment of the thermal detection thresholds (Suttrup et al., 2011). Furthermore, temporal judgment of somatosensory and auditory stimuli has been shown to be altered in musicians suffering from dystonia (Lim et al., 2003). On the other hand, cutaneous stimuli may reduce the severity of motor symptoms in some forms of focal dystonia. This phenomenon is termed *sensory trick*. Studies into the “geste antagoniste,” a sensory trick reducing severity of Torticollis, (a focal dystonia causing involuntary movements of the muscles of neck and shoulder) suggested that successful sensory tricks can be regarded as perceptual dysbalance, induce increased activation of the parietal cortex and together with the frontal cortex, mediates distinct sensorimotor transformations that can help with correcting the long-term dystonic posture. Accordingly, successful sensory tricks should be regarded as a complex dynamic mechanism that corrects the perceptual dysbalance of the abnormally defined posture (Naumann et al., 2000; Schramm et al., 2004). In task-specific focal dystonia, sensory stimuli like wearing a latex glove may also reduce the severity of motor symptoms in some forms of focal dystonia (Jabusch et al., 2011).

Based on the models proposed by the previous sensory trick studies, the present study aimed at investigating the effect of altered auditory feedback (AAF) on musician's dystonia (MD) under two widely used paradigms for studying motor behavior: (1) synchronization paradigm; (2) synchronization-continuation paradigm. The motivation for this study came from anecdotal reports of organ players suffering from hand dystonia who reported a marked improvement of the motor symptoms when playing on a pipe organ with delayed sound production after the key stroke due to mechanical coupling of the keyboard and the organ pipes. Such observation was valuable for the movement disorders research, yet the possible influence of AAF and altered somatosensory feedback on the fine motor control in MD had never been investigated. Considering the delayed auditory feedback in these reports, it is well known that the extensive training of professional musicians leads to plastic adaptations of neural networks involved in the demanding temporal-spatial control of overtrained movements (Bangert et al., 2006.; Zatorre et al., 2007), and it has been shown that AAF has a great influence on the motor control in music performance of healthy musicians (Pfordresher, 2006). Furthermore, it has been shown that AAF can induce improvement in speech in some movement disorders and motor speech disorders such as stuttering, dysarthria and Parkinson's disease (Downie et al., 1981; Gentil, 1993; Alm, 2004). Based on the above reasons, we therefore hypothesized that AAF could account for the anecdotal observations of the organ players and have an influence on the dystonic symptoms, similar to the cutaneous sensory trick in torticollis patients.

## Materials and methods

All procedures were approved by the ethics committee of Hannover Medical School and participants gave written informed consent before data collection. All patients suffering from MD were recruited from the outpatient clinic of the Institute of Music Physiology and Musicians' Medicine of the Hannover University of Music, Drama and Media. They underwent complete neurological examination and were diagnosed by one of the authors, who is a neurologist and movement disorders specialist (EA). All other neurological and health issues were excluded.

### Experiment 1: synchronization study

#### Participants

Twelve professional pianists (8 males, 4 females, mean age = 44.5, *SD* = 9.6, mean accumulated practice time = 42,086 h, *SD* = 25,040) suffering from right hand MD and 25 healthy professional pianists (13 males, 12 females, mean age = 25.8 years old, *SD* = 3.93, mean accumulated practice time = 25,075 h, *SD* = 10,754) participated in this experiment. Combined two one-sided *t*-tests (TOST, Robinson and Froese, 2004) shows that the two groups are not equivalent with regard of their accumulated practice hours (*p* = 0.92). According to the Edinburgh inventory (Oldfield, 1971), all the patients were right handed. Twenty four of the healthy professional pianists were right handed and one was left handed.

#### Procedure

The method is based on Scale Analysis (Jabusch et al., 2004). Subjects were instructed to repeatedly play 2 octaves of C major scales (from C4 to C6) in legato-style at a tempo of 80 beats per minute with four notes per beat (inter-onset intervals = 187.5 ms) in both upward and downward directions as accurately as possible on a MIDI digital piano (Wersi Digital Piano CT2) with their right hand only. There were three conditions for different types of auditory feedback: (1) *normal feedback* (NORMAL), in which the auditory feedback occurred simultaneously with the key depression; (2) *no feedback* (MUTE), in which no auditory feedback is produced with the key depression; (3) *fixed delayed feedback* of 200 ms (DELAY200), in which the auditory feedback occurred 200 ms after the key depression. The selection of delay duration was meant to mimick the delayed auditory feedback of a pipe organ in a resonant space. For each condition, the participant had to play at least 25 times of complete upward and downward scales. The order of the conditions was randomized, and all playing was synchronized to a real metronome placed on the digital piano (Wittner metronome QM2 taktell). In DELAY200, subjects were explicitly instructed to synchronize the piano sound to the metronome, not the movement. The onset and the offset time of each key depression was recorded (measured in milliseconds, start of the program is defined as 0 ms). MIDI recording, as well as manipulation for the conditions MUTE and DELAY, were done using a custom-made C program which acquired and (where applicable) manipulated the MIDI events coming from the MIDI piano. Sound was played back to subjects via the computer sound chip and two studio monitor speakers (Yamaha MSP 5) placed approximately 1 m in front of the subjects, 150 cm apart.

#### Data analysis

For each condition, as a measure of evenness of piano playing (which is required in professional pianists), the regularity of timing of successive keystrokes (termed *inter-onset intervals*, abbreviated as IOIs) was calculated from at least 20 sets of complete scales, except for the last IOI of every scale because it was frequently elongated according to the pianist's expressive playing. The coefficients of variation (CVs) were calculated to indicate the irregularity of timing of the scale playing. The CV is defined as: ${\hat{c}}_{v}^{*}=\left(1+\frac{1}{4n}\right)\frac{s}{x}$, where *s* is the standard deviation of the measured IOIs, *x* is the mean of the measured IOIs and *n* is the number of scales a participant played in a given condition (Sokal and Rohlf, 1995) and with this definition the term (1 + 1/4*n*) approaches 1 asymptotically with increasing *n*. The IOI CVs were computed for upward and downward scales individually since these are two different motor patterns. Finally the IOI CVs for all the patients and participants under all types of auditory feedback and for both upward and downward directions of scale playing were analyzed with ANOVA to detect the main effect of group and condition. The within factors are playing direction (with levels “up” and “down”) and types of auditory feedback (with levels “NORMAL,” “MUTE,” and “DELAY200”); the between factor is group (with levels “patient” and “control”). The means of IOIs for both playing directions under different conditions have been reported as well. All analyses were done using R (version 2.15.2; R Core Team, 2005) scripts in RStudio (version 0.97.551; RStudio, 2013). Multiple comparisons were corrected using Holm's (1979) method.

## Results of experiment 1

**Figure 1:**
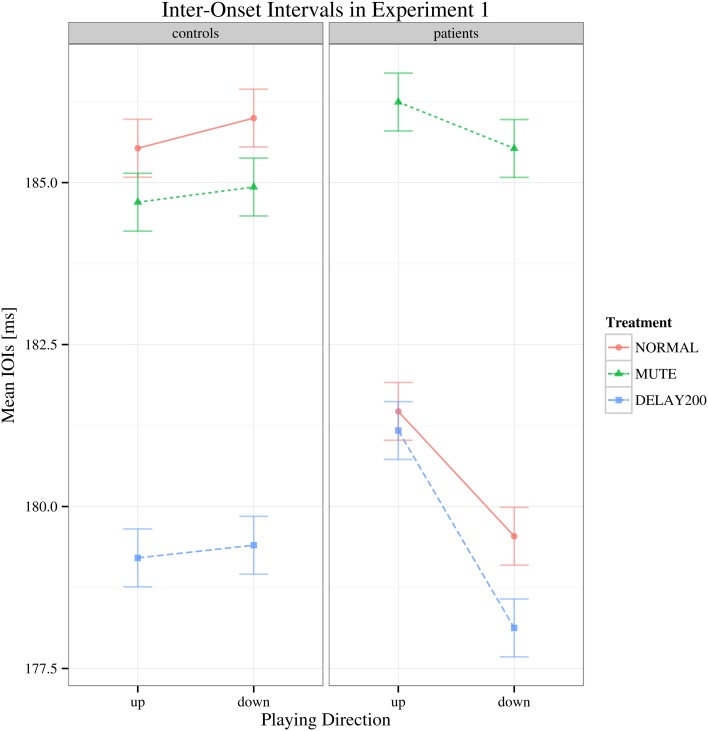
**Interaction plot of mean IOI's for Experiment 1.** Shown are the estimated mean IOIs (ms) conditional on group membership and auditory manipulation, collapsed over playing direction. Error bars denote 95% confidence intervals.

The target dependent variable in this study was the coefficient of variation of inter-onset intervals, being an objective measure of playing regularity in pianists with MD. To determine differences in playing speed, the inter-onset intervals were analyzed as well: regarding to the mean IOIs, a main effect of auditory feedback condition [*F*_(2, 70)_ = 8.832, Greenhouse-Geisser epsilon = 0.740, *p* < 0.001] was found but no effect of group [*F*_(1, 35)_ = 0.350, *p* > 0.05] or scale playing direction [*F*_(1, 35)_ = 2.445, *p* = 0.127] and no significant interactions were found [*F*_(2, 70)_ = 2.498, *p* > 0.05] (see Figure 1). The results of the healthy group during DELAY200 condition were untypical comparing to most reports of delayed auditory feedback. In our results of Experiment 1, the healthy group showed a decrease in mean IOIs (movement speeding up) while in most reports the participants show increased IOIs (movement slowing down). Possible explanations for this result are (1) as a strategy to cope with the delayed auditory feedback, the participants played faster in order to “get ahead” of the delay (Gates et al., 1974); (2) the effect of delayed auditory feedback on the timing of movement production is related to both the phase of actual movement execution and cognitive planning. It has been suggested that when delayed auditory feedback co-occurs with the downswing phase of movement execution, on a cognitive planning level, auditory information compliments the regulation of movement, and thus facilitates the approach to the goal, which may shorten the IOIs (Pfordresher and Dalla Bella, 2011). This effect might had played a role in our Experiment 1.

**Figure 2:**
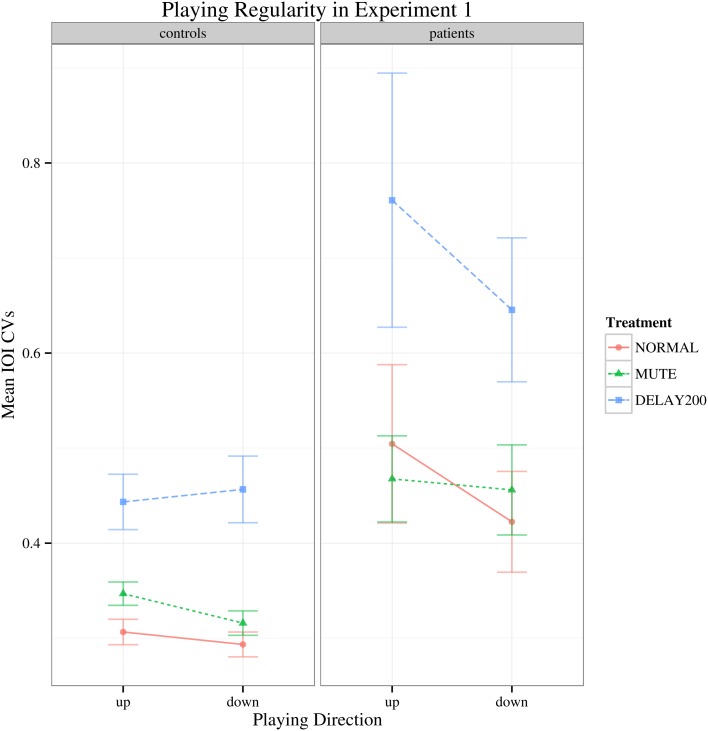
**Interaction plot of IOI CVs for Experiment 1.** Error bars denote 95% confidence intervals.

Regarding to the IOI CVs, there was a main effect of group [*F*_(1, 35)_ = 16.427, *p* < 0.001] and the patient group generally showed higher IOI CVs than the healthy controls. A main effect of feedback condition was also found [*F*_(2, 70)_ = 34.178, Greenhouse-Geisser epsilon = 0.676, *p* < 0.001], but no significant interactions were found (Figure 2). Both groups had increased IOI CVs for the DELAY200 condition, showing that DELAY200 induced unevenness in scale playing (pairwise *t-tests:* NORMAL-MUTE: *p* > 0.05; NORMAL-DELAY200: *p* < 0.001; DELAY200-MUTE: *p* < 0.001), meaning that they did not benefit from these two auditory manipulations in this synchronization paradigm. The patient group showed a very weak tendency toward higher IOI CVs in upward scale playing [Figure 2; *F*_(1, 35)_ = 3.497, *p* = 0.07]. This is possibly related to the task-specificity of focal hand dystonia revealed under different motor patterns involved in playing directions. It should be mentioned, that the upwards scales are more complex due to the thumb-under passage, requiring a complex anticipatory coordination maneuver of thumb, remaining four fingers and the wrist.

### Discussion of experiment 1

The results of Experiment 1 showed that neither did the complete deprivation of auditory feedback nor did a constantly delayed auditory feedback of 200 ms serve as a successful sensory trick, which is against our hypothesis. Experiment 1 also showed an effect of auditory feedback condition on mean IOIs in both groups. However, the two groups of participants are very different in terms of their age and their years of accumulated hours of practice. Pianists suffering from MD are generally older than the healthy controls. In Experiment 2, we had tried our best to reduce this discrepancy.

### Experiment 2: synchronization-continuation study

Following Experiment 1, in which deprivation of auditory feedback and delayed auditory feedback did not serve as a sensory trick that could improve the fine motor control in MD, Experiment 2 was carried out. One problem inherent to playing scales with delayed feedback to a metronome is probably the higher task difficulty in this condition. Anticipating the auditory feedback and executing the key presses 200 ms in advance during scale playing in order to exactly synchronize with the metronome proved to be difficult for both healthy pianists and pianists suffering from MD, and this might have shifted the pianist's attention away from the scale playing toward the out-of-phase synchronization of movements to the metronome, leading to a different neural process that is required to pace the movement according to an external stimulus (Serrien, 2008). To circumvent this problem, in Experiment 2 we applied the synchronization-continuation paradigm, and reduced the delay to 90 ms since this would reduce the degree of asynchrony involved in Experiment 1.

#### Participants

Twelve professional pianists suffering from right hand MD (9 males, 3 females, mean age = 41, *SD* = 10.4, mean accumulated practice time = 37,593 h, *SD* = 11199) and 12 healthy professional pianists (5 males, 7 females, mean age = 33.3 years old, *SD* = 11.6, mean accumulated practice time = 35,495 h, *SD* = 24,317) participated in this experiment. TOST suggested that the two groups were not equivalent with regard of their accumulated practice hours (*p* = 0.356). All the pianists suffering from MD were right handed. Eleven of the healthy controls were right handed and 1 was left handed, according to the Edinburgh inventory (Oldfield, 1971). Two of the pianists suffering from right hand MD had participated in Experiment 1. None of the healthy pianists had participated in Experiment 1.

#### Procedure

Similar to Experiment 1, the method of Experiment 2 is also based on Scale Analysis, but the conditions are designed according to the synchronization-continuation paradigm (Stevens, 1886; Finney and Warren, 2002; Pfordresher and Palmer, 2002), and one more condition, which is the altered tactile feedback condition, was included. The synchronization-continuation paradigm is widely used in studies of temporal coordination between actions and sound. During the experiment, the participants were instructed to repeatedly play 2 octaves of C major scales (from C4 to C6) with right hand in legato-style at a tempo of 80 beats per minute with 16th notes (four notes per beat, inter-onset interval = 187.5 ms) in either upward or downward directions as accurate as possible on the same digital piano used in Experiment 1. The first phase of each condition was the synchronization part, the participant had to play 10 sets of scales according to a metronome click sound generated by the computer and played through two speakers (Yamaha MSP 5). The second phase of each condition was the continuation part and the participant had to maintain the same way and same tempo of playing for at least 20 sets of complete and error-free scales in the absence of metronome sound. It was in the continuation part that the AAF (if any) was employed. Similar to Experiment 1, there were three different types of auditory feedback used in the continuation part: (1) normal auditory feedback (NORMAL); (2) muted feedback (MUTE); (3) with fixed delayed feedback of 90 ms (DELAY90). In the (4) tactile condition, auditory feedback was as in NORMAL but subjects wore a latex glove (GLOVE) on their playing hand throughout both the synchronization and continuation phases. The order of the conditions was randomized in two blocks. In one block the participants played upward scales only, and in the other block they played downward scales only. The order of the two blocks was also randomized. Between the blocks the participants were instructed to take a 2 min rest. The metronome sound onset time in the synchronization phase and the onset and the offset time of each key depression were recorded (measured in milliseconds, start of the program is defined as 0 ms). Other than that, apparatus was the same as in Experiment 1.

#### Data analysis

Both the synchronization and the continuation parts of each condition were analyzed. The first scale of the continuation phase was discarded from the analysis because of the common increase in unevenness among the participants. Similar to Experiment 1, the IOI CVs from at least 20 sets of complete, error-free scales were calculated to indicate the irregularity of timing of the participants in each condition. To determine how well subjects synchronized with the metronome, we ran a within-between ANOVA on the mean temporal deviation of key presses from the metronome tick [between factor: group (levels “patient” and “control”); within-factors: treatment (levels “NORMAL,” “MUTE,” “DELAY90,” “GLOVE”) and playing direction (levels “up,” “down”)]. On the IOI CVs, again a mixed effects ANOVA was calculated (factors and levels same as just mentioned). After the results suggested that there was no effect of playing direction, a between-within Ss ANOVA was run on the mean IOI CVs collapsed across playing directions to increase possible group effects [between effect: group (levels “control,” “patient”), within effect: treatment (levels “NORMAL,” “MUTE,” “DELAY90,” “GLOVE”)]. All analyses were done using R (version 2.15.2; R Core Team, 2005) scripts in RStudio (version 0.97.551; RStudio, 2013).

## Results of experiment 2

**Figure 3:**
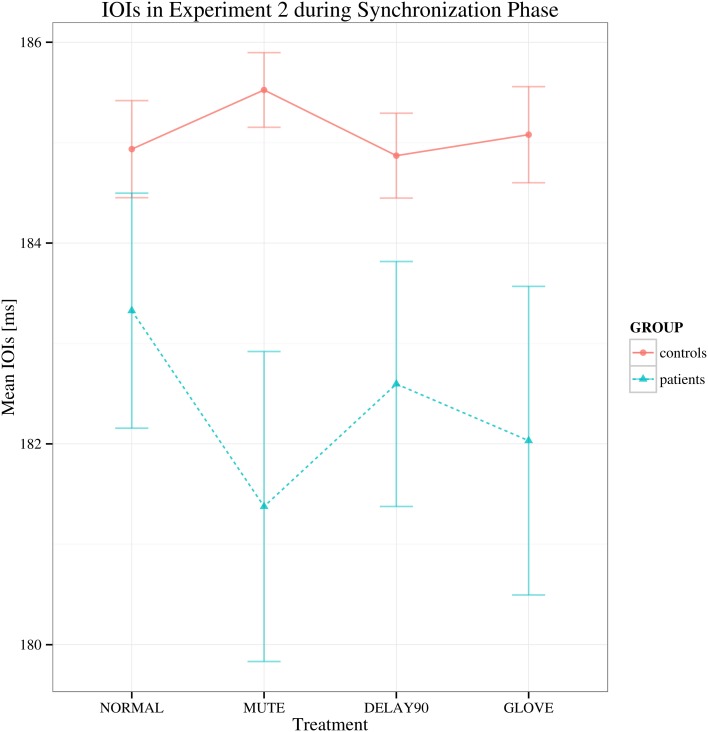
**Mean of the mean deviations from the metronome in the synchronization phase in Experiment 2.** Shown are the estimated mean IOIs (ms) conditional on group membership and auditory manipulation, collapsed over playing directions. Error bars denote 95% confidence intervals.

For the synchronization phase, both groups display negative asynchrony (keypress was in advance of the metronome; Repp, 1999). No significant main effect of group was found in this phase, suggesting that there is no difference between the patient and control group in terms of synchronization [*F*_(1, 22)_ = 2.679, *p* > 0.05; Figure 3], showing that both groups had similar degree of synchronization to the metronome. There were also no statistically significant effects of sensory feedback [*F*_(3, 66)_ = 0.877, Greenhouse-Geisser epsilon = 0.513, *p* > 0.05] and playing direction [*F*_(1, 22)_ = 2.807, *p* > 0.05].

**Figure 4:**
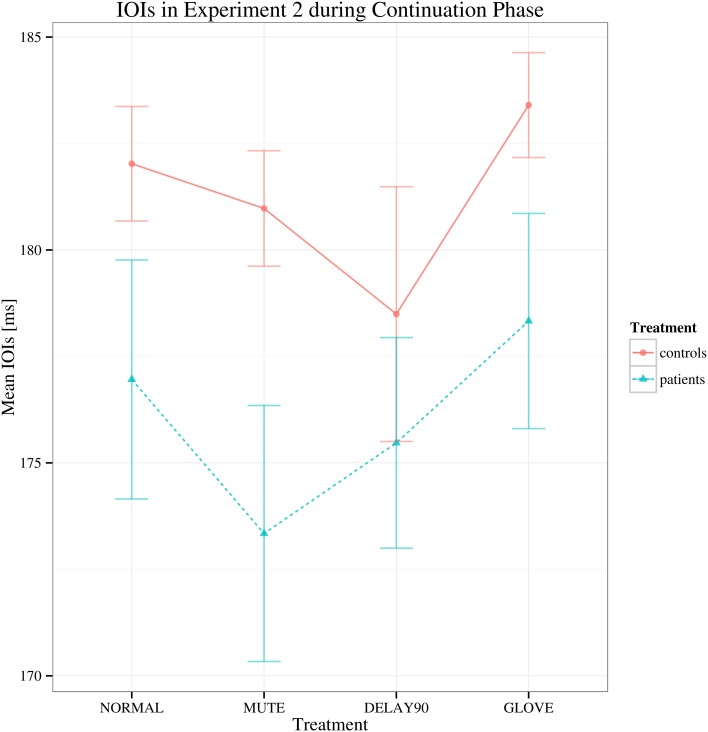
**Interaction plot of mean IOIs for Experiment 2 during continuation phase.** Details as in Figure 1. Error bars denote 95% confidence intervals.

For the continuation phase, regarding to the mean IOI's, no main effects and no significant interactions were found [group: *F*_(1, 22)_ = 1.726, *p* > 0.05; sensory feedback: *F*_(3, 66)_ = 3.069, Greenhouse-Geisser epsilon = 0.529, *p* > 0.05; playing direction: *F*_(1, 22)_ = 0.001, *p* > 0.05; group × sensory feedback: *F*_(3, 66)_ = 0.772, *p* > 0.05) (Figure 4).

**Figure 5:**
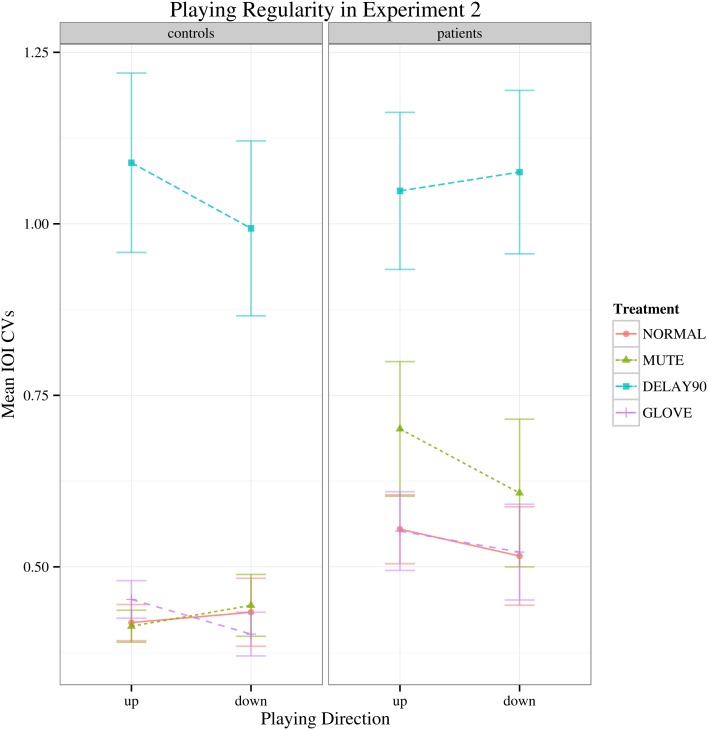
**Interaction plot of IOI CVs for the continuation phase of Experiment 2.** Error bars denote 95% confidence intervals.

Similar to Experiment 1, with regard to the IOI CVs, a significant main effect was found for sensory feedback [*F*_(3, 66)_ = 93.026, Greenhouse-Geisser epsilon = 0.385, *p* < 0.001], possibly attributable to a significantly deteriorated motor performance for DELAY condition during the continuation phase (pairwise *t*-tests: NORMAL-MUTE: *p* > 0.05; NORMAL-DELAY90: *p* «0.05; NORMAL-GLOVE: *p*>0.05; Figure 5). No significant interactions were found [*F*_(3, 66)_ = 1.65, *p* > 0.05]. Individual mean IOI CVs of *all the patients* during the continuation phase under different conditions are detailed in Table 1, with the IOI CVs of upward and downward directions averaged. From the table, one can see that only four patients who have less severe symptoms had slightly improved motor control under MUTE and only one patient had improved motor control under DELAY90 condition, while five patients had slightly improved motor control under GLOVE condition. Improved IOI CVs are marked in bold and italic fonts. Paired and one-sided *t*-tests of the IOI CVs calculated from each scale run were used to verify if the patient had significantly improved motor control under certain condition. For each patient, the NORMAL condition is tested against other altered sensory feedback conditions. The alternative hypothesis is that the true difference between the means is greater than 0. Therefore, in Table 1, the altered sensory feedback conditions that show significantly reduced IOI CVs are the ones having *p* < 0.05 and are underlined.

**Table 1:** **Individual mean IOI CVs of all the patients in Experiment 2**.

**Patient ID**	**Normal**	**Mute**	**Delay90**	**Glove**
p01	0.63	0.635	1.245	***0.595***
p02	0.29	***0.275***	1.005	0.335
p03	0.315	0.485	0.855	0.32
p04	0.435	0.465	0.955	0.49
p05	0.445	0.61	1	0.45
p06	0.315	***0.28***	0.785	***0.285***
p07	0.57	0.63	0.92	0.61
p08	0.495	***0.36***	0.895	***0.355***
p09	0.62	0.825	1.445	*0.57*
p10	0.335	0.34	0.84	*0.315*
p11	0.275	***0.255***	***0.21***	*0.27*
p12	0.445	0.5	0.715	***0.39***

The conditions in which the patient showed less IOI CVs are marked with bold and italic fonts, and are underlined if p < 0.05 according to the paired and one-sided t-tests.

Compared to Experiment 1, both the healthy controls and the patients had markedly increased IOI CVs for the delay condition. This might be due to the effects of combined delayed auditory feedback plus pitch alteration are smaller than either kind of alteration on its own (Pfordresher, 2003). In Experiment 1, the duration of delay was 200 ms, which was longer than the theoretical IOI of keypresses (187.5 ms), induced pitch shift relative to the keypress as well; whereas in Experiment 2, the duration of delay was 90 ms, which was shorter than the theoretical IOI of keypresses, did not induce such pitch shift. Interestingly, in Experiment 2, there was no main effect of group while in Experiment 1, there was significant main effect of group. This stark contrast might be due to the different durations of the delay (200 ms for Experiment 1 and 90 ms for Experiment 2) and the different nature of the tasks (synchronization in Experiment 1 and continuation phase in Experiment 2). Playing with delayed auditory feedback in the continuation phase was much more difficult than in the synchronization phase, the healthy subjects showed significantly deteriorated fine motor control as well therefore there was no clear main effect of group.

## Discussion

In both Experiments 1 and 2, we found no difference between the behavior of healthy controls and patients. None of the AAF that we employed (either MUTE or DELAY of 200 and 90 ms), and nor did the altered tactile feedback (GLOVE) improve the fine motor control of pianists suffering from MD. Taken together, the results are against our hypothesis, which assumed that the AAF or altered tactile feedback might serve as forms of successful sensory trick.

To the best of our knowledge, the only study that tested the role of auditory feedback in dystonia is a case study (Kojovic et al., 2012) in which a patient with generalized DYT1 dystonia showed dramatically improved symptoms while playing electric piano with auditory feedback. This improvement was reduced while the auditory feedback was masked but still noticeable. However, the reduction of dystonic symptoms in this case is considered different from typical sensory tricks, and is proposed to be more similar to *paradoxical improvement* (Fahn, 1989). What's more, primary generalized dystonia and focal dystonia differ in many respects, including the thresholds of sensory spatial discrimination (Molloy et al., 2003).

AAF has been extensively studied in speech and music performance related research. It has been shown that stuttering frequency can be significantly decreased by masking the auditory feedback (e.g., MacCulloch et al., 1970) or by delayed auditory feedback (e.g., Kalinowski et al., 1996), and delayed auditory feedback may serve as a treatment (Van Borsel et al., 2003). In music performance, auditory feedback has been shown to play a crucial role in the self-monitoring of music performance. It is shown that AAF can profoundly disrupt the performance of healthy professional musicians, and asynchronies between auditory feedback and actions primarily disrupt the timing of actions (Pfordresher, 2006). In the present study, AAF, especially delayed auditory feedback, disrupted the performance of both healthy pianists and pianists suffering from MD.

The MUTE condition can be seen as a task which directs the participants' attention toward their own movement. Theoretically speaking, the deprivation of auditory feedback would de-automatize the extensively trained auditory-motor coupling of expert musicians, thus recruit the structures outside the basal ganglia—supplementary motor area (SMA) system, such as the premotor cortex, which can only provide internally cued complex motor sequence during de-automatization (Alm, 2004). Nevertheless, only a few patients who had less severe symptoms participated in the current study showed mild benefit from such attentional shift, suggesting that the abnormal neural network involved in MD may not be normalized by this de-automatization, and it is reasonable to speculate that the more severe MD is more closely linked to deficient procedural memory, as is the case for several other movement disorders (Doyon, 2008). Although it has been shown that the functional cortical network in focal hand dystonia patients is impaired (Jin et al., 2011), the deprivation of auditory feedback in the long-trained coupling of sensorimotor cortical areas did not act as successful sensory trick for our patients. Instead, it has even been shown to have a detrimental effect on MD patient's fine motor control in the present study. The possible explanation for this finding is that playing scales under the MUTE condition relies heavily on an internal model that does not require auditory feedback, and the internal model has been shown to be impaired in MD (Ruiz et al., 2009, 2011; Lee et al., 2013).

The DELAY condition is essentially a task which manipulates the effect produced by participants' movements, which is similar to the studies that investigated the influence of the performer's focus of attention, which suggested that directing one's attention to the effects of the movements (external focus) involves different motor control processes than directing one's attention to one's own movements (internal focus) (McNevin et al., 2003). In case of movement disorders, it has been shown that patients with Parkinson's disease and a fall history can improve their balance with the adoption of an external focus (Landers et al., 2005). It should be noted that the sensory trick was once considered as a manoeuvre for distracting patients' attention (Abbruzzese and Berardelli, 2003). Nevertheless, in the current study, it is also shown that the manipulation of an external focus deteriorates the motor output and does not bring any benefit to the patients the way a successful sensory trick may do. It is interesting to note that the patients' playing was still affected by the manipulation of the external focus of attention similar to the healthy controls, implying the action and sound production are still strongly coupled in MD patients.

One of the studies that put an emphasis on the altered tactile feedback is a study carried out by Jabusch et al., (2011). Nevertheless, in this study, the authors addressed a different research question which aimed at the potential association between tactile sensory trick phenomenon (“glove effect” in this study) and the outcome after consequent treatment with botulinum toxin and/or pedagogical re-training. Furthermore, in this previous study, only 19% of patients showed significant improvement of fine motor control through wearing a glove in this study, and the patients who participated in this previous study had more severe symptoms (with median of standard deviations of inter-onset intervals of scale playing = 20.0 ms) than our patients (median of standard deviations of inter-onset intervals of scale playing = 14.76 ms). In the present study, we have smaller samples sizes (12 patients in both experiments) than the previous study. Both differences (degree of severity and sample size) could explain why in the current study there were no effects of glove on a group level. It should be mentioned, however, in the present study several individuals suffering from focal dystonia benefitted from glove condition. This points toward the heterogeneity in neural and/or behavioral organization of MD.

Behavioral studies on the sensory functions in dystonia is gaining its importance in light of providing effective strategies for recovery and understanding the underlying mechanisms (Tinazzi et al., 2009). In MD, although it has been shown that patients have deficits in temporal judgment, it was not clear how this sensory deficit can affect their playing ability under a disrupted sensory feedback network. Although the starting point of this study was the reports related to pipe organs having delayed auditory feedback, the present study provides evidence showing that the defected sensory network could not be normalized by the AAF conditions used in this experiment or altered tactile feedback such as wearing the glove. We hope that these results can contribute into further research in the sensory aspects of focal task-specific dystonia and help us to provide the patients with correct information and guide the patients with the most efficient therapeutic options.

### Conflict of interest statement

The authors declare that the research was conducted in the absence of any commercial or financial relationships that could be construed as a potential conflict of interest.
